# Characteristics of Multi-Organ Lymphangiectasia Resulting from Temporal Deletion of *Calcitonin Receptor-Like Receptor* in Adult Mice

**DOI:** 10.1371/journal.pone.0045261

**Published:** 2012-09-17

**Authors:** Samantha L. Hoopes, Helen H. Willcockson, Kathleen M. Caron

**Affiliations:** 1 Department of Cell Biology and Physiology, University of North Carolina, Chapel Hill, North Carolina, United States of America; 2 Department of Genetics, University of North Carolina, Chapel Hill, North Carolina, United States of America; National Institutes of Health, United States of America

## Abstract

Adrenomedullin (AM) and its receptor complexes, *calcitonin receptor-like receptor (Calcrl)* and *receptor activity modifying protein 2/3*, are highly expressed in lymphatic endothelial cells and are required for embryonic lymphatic development. To determine the role of *Calcrl* in adulthood, we used an inducible Cre-loxP system to temporally and ubiquitously delete *Calcrl* in adult mice. Following tamoxifen injection, *Calcrl^fl/fl^/CAGGCre-ER™* mice rapidly developed corneal edema and inflammation that was preceded by and persistently associated with dilated corneoscleral lymphatics. Lacteals and submucosal lymphatic capillaries of the intestine were also dilated, while mesenteric collecting lymphatics failed to properly transport chyle after an acute Western Diet, culminating in chronic failure of *Calcrl^fl/fl^/CAGGCre-ER™* mice to gain weight. Dermal lymphatic capillaries were also dilated and chronic edema challenge confirmed significant and prolonged dermal lymphatic insufficiency. *In vivo* and *in vitro* imaging of lymphatics with either genetic or pharmacologic inhibition of AM signaling revealed markedly disorganized lymphatic junctional proteins ZO-1 and VE-cadherin. The maintenance of AM signaling during adulthood is required for preserving normal lymphatic permeability and function. Collectively, these studies reveal a spectrum of lymphatic defects in adult *Calcrl^fl/fl^/CAGGCre-ER™* mice that closely recapitulate the clinical symptoms of patients with corneal, intestinal and peripheral lymphangiectasia.

## Introduction

The lymphatic vascular system is a complex vascular network that permeates nearly every organ of the body and plays a critical role in the maintenance of fluid homeostasis, the absorption of intestinal lipids and the trafficking and maturation of immune cells [1]. Despite its pervasive functions, it is surprising that very little is known about the genetic and molecular pathways that regulate lymphatic vascular function in adults [2]. Fortunately, the past dozen years has provided a relative explosion of new and sometimes unexpected genes involved in the development of the lymphatic vascular system, based largely on elegant and exciting embryonic phenotypes uncovered in gene knockout studies in mice and in vertebrate model organisms like zebrafish and xenopus [3]. Some of these discoveries have even paved the way toward the identification and better understanding of human genes in which mutations are causally associated with congenital, primary lymphedema such as *FOXC2*, *FLT4, SOX18, GJC2* and *CCBE1*. Nevertheless, the development of additional genetic mouse models of lymphatic insufficiency during adulthood is still needed in order to identify novel candidate genes either for genetic testing in families with congenital forms of lymphedema or for therapeutic targeting of lymphatics in disease.

Failure of lymphatic vessels to function properly in adults can result in numerous types of clinical conditions, including primary and secondary lymphedema, which can have a broad range of clinical presentations and associated correlates [4], [5]. Some congenital forms of primary lymphedema are associated with lymphangiectasia, which is typically characterized as dilation and enlargement of lymphatic vessels. Interestingly, there are a few organ systems, including the intestine [6], the conjunctiva of the eye [7] and the dermis [8], that are particularly prone to developing lymphangiectasia. While the pathophysiological mechanisms leading to lymphangiectasia are not well understood, it is likely that dilated lymphatic vessels are the result of lymphatic obstruction and improper drainage or lymph stasis. The consequences of persistent lymphangiectasia include, on a cellular level, increased permeability of dilated lymphatic vascular beds, and on a systemic level, protein-losing enteropathy, limb lymphedema and ocular irritation with dryness. Although lymphangiectasia can be associated with a variety primary, congenital lymphedema syndromes, there is currently no known genetic pathway that directly and predominantly contributes to lymphangiectasia.

Using gene targeting approaches in mice, we have previously discovered and characterized an essential role for adrenomedullin (gene = *Adm*, peptide = AM) peptide and its receptor complex in lymphatic vascular development. Adrenomedullin, a secreted, multi-functional peptide that is highly expressed in endothelial cells, binds and signals through a G protein-coupled receptor, *calcitonin receptor-like receptor (*gene = *Calcrl;* protein = CLR*)*, when the receptor is associated with receptor activity modifying proteins 2 or 3 (RAMP2/3*).* The complex formed by CLR and RAMP2 is referred to as the adrenomedullin 1 (AM1) receptor, while the CLR and RAMP3 complex is referred to as the AM2 receptor; both of which bind AM peptide, but differ in their relative binding affinities [9]. Gene knockout mice for *Adm* [10], *Calcrl* [11], and *Ramp2* [12], [13] all exhibit mid-gestational embryonic lethality characterized by hydrops fetalis, or marked edema, that is associated with arrested lymphatic vascular development. Conditional deletion of *Calcrl* in endothelial cells confirmed that AM signaling, and its downstream activation of the MAPK/ERK signaling cascade, is required for normal lymphatic endothelial cell proliferation during development.

AM signaling through *Calcrl*/*Ramp2* also has robust effects on endothelial cell permeability. For example, AM can abrogate the permeabilizing effects of hydrogen peroxide and thrombin on human umbilical vein endothelial cells [14] and it can retard the transport of molecules across the blood brain barrier by tightening the permeability of cerebral endothelial cells [15], [16]. Similarly, we have shown that AM can impact the permeability and function of lymphatic endothelial cells (LECs). Treatment of cultured LECs with AM significantly and functionally reduced their permeability by causing a subcellular reorganization of the junctional proteins ZO-1 and VE-Cadherin [17]. Furthermore, *in vivo* tail microlymphography reinforced these findings since mice injected with AM showed reduced lymph velocity through dermal lymphatic capillaries, indicative of functionally reduced permeability [17].

The apparently biased effects of AM signaling on the embryonic development of lymphatic vessels, versus blood vessels, is likely attributable to the increased expression of *Calcrl* and *Ramp2* in LECs, compared to blood endothelial cells [18]–[20]. Consistent with this notion, continuous administration of AM promoted lymphangiogenesis and ameliorated secondary tail lymphedema in a surgical injury mouse model [21]. Whether the maintained expression of *Calcrl* in adult animals is also required for appropriate lymphatic function remains unclear. To address this question, we used a ubiquitously expressed, tamoxifen-inducible Cre transgenic mouse line (*CAGGCre-ER™*) to delete a floxed *Calcrl* gene in 3–4 month old animals and thus explore the role of *Calcrl* during adulthood. Our results continue to support a preferential role for *Calcrl* in the lymphatic vasculature and reveal that *Calcrl* expression in adult animals is critical for maintaining the proper function of lymphatic vessels in a wide variety of organs.

## Methods

### Animals

Mice used in these studies were generated from crossing *Calcrl^fl/fl^* [12] mice (N7-10 on C57BL/6 background) to *CAGGCre-ER™* mice [The Jackson Laboratory, Bar Harbor, ME 004682, B6.Cg-Tg(CAG-cre/Esr1)5Amc/J]. Male and female adult mice aged 3–4 months were administered tamoxifen (Sigma) consecutively for 5 days (5 mg/40 g body weight; IP). Mice were genotyped for the floxed and Cre alleles as well as the excised allele after tamoxifen injection. Primer sets (5′-3′) P1: gcggagcatattcaatcacaag, P2: gaaatgtgctgtatgttcaagc, P3: gacgagttcttctgagggga, and P4: gaataagttgagctgggcag were used (P1/P2 for wildtype allele; P1/P3 for floxed allele; P1/P4 for excised allele). Mice were routinely anesthetized using 0.2–0.4 ml/10 g body weight of avertin (2,2,2,-Tribromoethanol, 20 mg/ml, Sigma).

For Western Diet studies, mice were fed Teklad Adjusted Calories Diet (TD.88137; 42% from fat; Harlan Laboratories) for 1½ weeks and then housed in metabolic cages for 24 hours during which food intake, urine, and fecal samples were measured. Weights of mice were also recorded before tamoxifen injection, after tamoxifen injection, and after Western Diet.

All experimental procedures involving mice were approved by the Institutional Animal Care and Use Committee of The University of North Carolina Chapel Hill and all efforts were made to minimize suffering.

### Cell Culture

Human adult dermal lymphatic endothelial cells (HMVEC-dLyAd-Der Lym Endo Cells, Lonza) of 8 passages or less were maintained using EGM-2MV media with bullet kit (Lonza). Cells were seeded in 6 well plates at 100,000 cells/well and grown on acid washed coverslips until monolayers formed. Treatment conditions included no treatment (control), 10 nM AM (American Peptide Co.,Inc.), 1 µM AM22-52 (AM antagonist; American Peptide Co.,Inc.) or AM+AM22-52. Cells were treated for 15 minutes and in the condition with AM+AM22-52, cells were pre-treated with AM22-52 for 30 minutes. Cells were rinsed with HBSS, fixed with 1% PFA, rinsed 3×5 minutes with PBS, and then blocked for 20 minutes with 2% normal donkey serum/0.1% Triton X in PBS. The cells were then incubated overnight at room temperature with primary antibodies (VE-Cadherin = 1∶200, goat polyclonal; sc-6458, Santa Cruz Biotechnology, Santa Cruz, CA; ZO-1 = 1∶200, monoclonal rat α mouse; clone R40.76, Millipore, Billerica, MA), rinsed 3×5 minutes with PBS, blocked with 2% normal donkey serum (NDS) for 10 minutes, followed by incubation with secondary antibody for 1 hour at room temperature, rinsed 3×5 minutes with PBS and then mounted on slides using Mowiol.

### Immunohistochemistry and Immunofluorescence

Tissues were dissected, fixed with 4% PFA overnight and embedded in paraffin or protected in 30% sucrose and embedded in OCT (Tissue-Tek) for sectioning. Sections were permeabilized using 0.1% Triton X-100 (in 0.01 M PBS; pH 7.2; 15 minutes), blocked with 5% NDS (in 0.1% Triton X-100; 30 minutes), incubated overnight in primary antibodies, PBS rinsed (3×5 minutes), blocked with 5% NDS (30 minutes), incubated with secondary antibodies (2 hours), rinsed with PBS and coverslipped with Mowiol. Primary antibodies included: LYVE-1 (1∶200; polyclonal rabbit α mouse; Fitzgerald, Acton, MA), podoplanin (1∶200, Syrian hamster α mouse, Developmental Studies Hybridoma Bank, Univ. Iowa), ZO-1 (1∶200, monoclonal rat α mouse; clone R40.76, Millipore, Billerica, MA) and VE-Cadherin (1∶200, goat polyclonal; sc-6458, Santa Cruz Biotechnology, Santa Cruz, CA). Secondary antibodies included Alexa Fluor 594, Alexa Fluor 488 and Cy3 (1∶200, Jackson Immunoresearch) and nuclear marker DAPI (1∶1000, bisbenzimide 33258; Sigma, St. Louis, MO). TUNEL staining was performed using the ApopTag Fluorescein *In Situ* Apoptosis Detection Kit (S7110, Chemicon International) according to the manufacturer’s protocol.

### Tonometry

Tonometry was performed in anesthetized adult mice using a TonoLab tonometer (Colonial Medical Supply) as described previously [22], [23]. After avertin injection, a drop of tetracaine hydrochloride 0.5% (Alcon) was placed on the eye as a local anesthetic. Eyes were lubricated throughout testing with TEARS Naturale FORTE (Alcon). At least six readings were recorded per eye and averaged.

### Tail Microlymphography and Vessel Diameter

Three to four months post tamoxifen injection, adult mice were used for tail microlymphography as described previously [17] with several modifications. FITC-conjugated dextran (200 kDa; 1 µl; Molecular Probes, Invitrogen Detection Technologies) was injected intradermally into the mouse tail using a 5 µl Hamilton syringe fitted with a 30 gauge needle. Images were taken every minute for 15 minutes and image analysis was performed using Adobe Photoshop 7.0 and Image J.

### Lymphatic and Blood Permeability Assays

An ear lymphatic permeability assay was performed as previously described [24] with minor modification. Ears of anesthetized mice were injected intradermally with 2 µl of 0.5% Evan’s Blue dye (in saline) with a 10 ul Hamilton syringe. Images were taken immediately after injection and 5 minutes after injection. A blood permeability assay was performed as previously described with slight modifications to the protocol [25]. Anesthetized mice were retro-orbitally injected with 200 µl 0.5% Evan’s Blue dye (in saline). After 30 minutes, the mice were perfused with saline and the liver, lung, adductor muscle, spleen, intestine, heart, and brain were harvested. Tissues were weighed and desiccated overnight at 55°C followed by formamide extraction (55°C, overnight) and 100 µl was used for absorbance reading at 600 nm.

### Acid Steatocrit/Lipase/Triglyceride Measurements

Fecal samples collected after Western Diet were examined by testing for fecal steatocrit and fecal lipase as previously described [26] with recent modifications [27]. Fecal specimens were powdered and mixed with 1N perchloric acid and 0.5% oil red O and placed in a capillary tube and centrifuged. Steatocrit was calculated as 100 × [length of fatty layer/(length of solid layer+length of fatty layer)]. Fecal lipase and serum triglycerides were analyzed at the Animal Clinical Chemistry and Gene Expression Labs (UNC-CH).

### Dot Blot Assay

A dot blot assay using digested fecal samples was performed as previously described [28] with slight modification. In short, mice were fed Western Diet for 1½ weeks and fecal samples were collected and stored at −20°C. TBS with 5% nonfat dry milk (with protease inhibitors) was added to the fecal samples (20 µl/mg). The samples were vortexed and sonicated then centrifuged at 16,000 g for 10 minutes after which the supernatant was collected. Three microliters of each supernatant (1∶2000 dilution) was dotted onto a nitrocellulose membrane. The membrane was blocked (TBS+3% nonfat dry milk) for 2 hours at room temperature, rinsed with TBST (1×5 minutes), then incubated overnight with primary antibody (mouse anti alpha-1 antitrypsin-1∶500; Novus and mouse anti-actin-1∶10000; Sigma) in TBST+3% nonfat dry milk at 4°C. The membrane was rinsed with TBST (3×10 minutes), incubated with secondary antibody in TBST (HRP goat anti-mouse; 1∶2000; Upstate) for 45 minutes at room temperature followed by TBST (3×5 minutes) rinses and a final TBS (1×5 minutes) rinse. The membrane was then developed with film (GeneMate) using WesternBright ECL reagents (Advansta). Analysis of integrated density was performed using Image J.

### Edema Formation Assay

Anesthetized mice were injected with 10 µl of 4 µg/µl Complete Freund’s Adjuvant (CFA) in one hind paw. The other hind paw served as an internal control. Paw thickness was measured with calipers before injection of CFA and every other day after injection up to 21 days.

### RNA and qRT-PCR

Lung and heart tissue were collected in RNAlater Solution (Ambion). RNA was extracted from tissue using TRIZOL (Ambion, Life Technologies) isolation followed by DNAse treatment (Promega) and cDNA preparation. Quantative RT-PCR was performed on a Stratagene Mx-3000p machine (La Jolla, CA) using TAQMAN GEx Master Mix (Applied Biosystems). *Calcrl* expression was assessed using Assay-on-Demand for *Calcrl* (Mm00516986_m1; Applied Biosystems). The comparative quantitation (ΔΔC_T_) method was used to determine the relative level of *Calcrl* expression in the tissues compared to mouse embryo total RNA calibrator (Ambion). All assays were repeated at least three times and run in duplicate.

### Statistical Analysis

All experiments were repeated at least 3 times and data are expressed as means with SEM values. Student *t* tests (tails = 2, type = 3) and two-way ANOVA were performed and P≤0.05 was considered significant.

## Results

### Temporal Deletion of *Calcrl* Results in Acute Onset Eye Phenotype with Enlarged Corneoscleral Lymphatic Vessels

**Figure 1:**
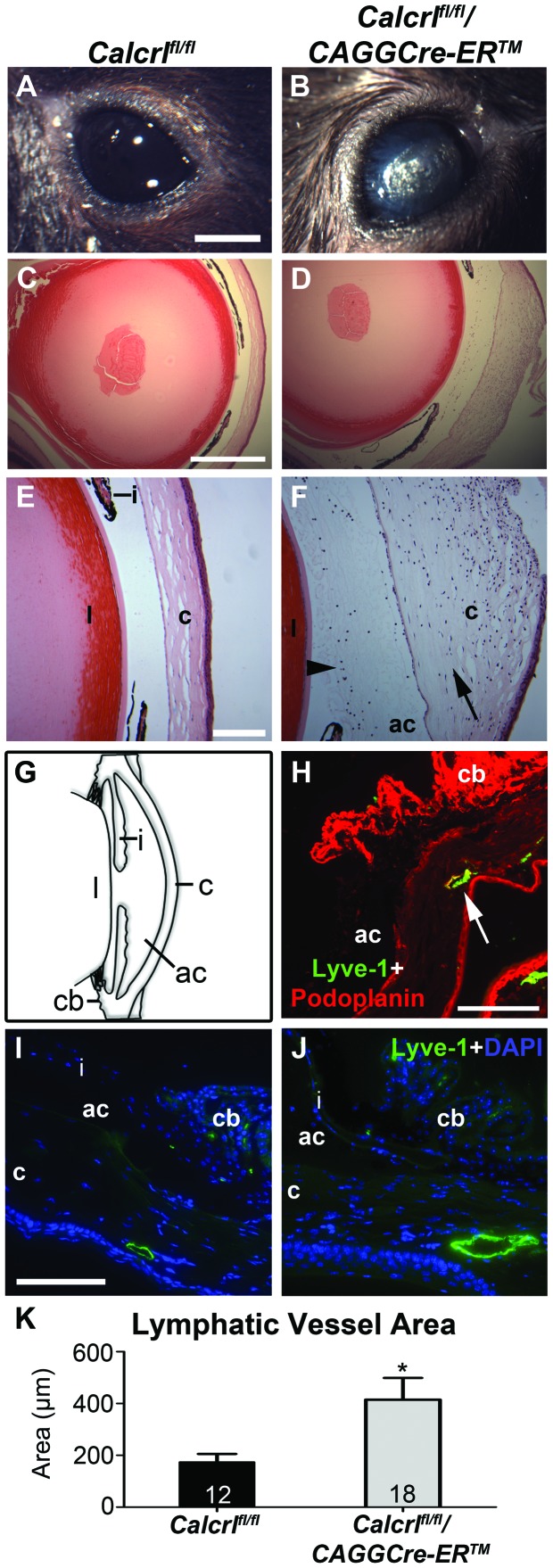
Acute-onset eye phenotype, eye inflammation, edema, and enlarged lymphatic vessels in *Calcrl^fl/fl^/CAGGCre-ER™* mice. A,B, Gross eye images indicating normal appearance of the control *Calcrl^fl/fl^* mice (A) and the distinct color change and disruption of the cornea of *Calcrl^fl/fl^/CAGGCre-ER™* (B), (scale = 2 mm). **C,D,** Hematoxylin and eosin staining of mouse eyes indicating normal histology in *Calcrl^fl/fl^* (C) and disruption of the cornea in *Calcrl^fl/fl^/CAGG-CreER™* mice (D), (4x objective,scale = 500 µm). **E,F,** Higher magnification of histological sections of eyes from *Calcrl^flf/fl^* mice (E) as compared to *Calcrl^fl/fl^/CAGGCre-ER™* mice (F) exhibiting corneal edema (arrow) and inflammation (arrowhead) (10x objective, scale = 200 µm). Gross anatomy and histology images are representative from *Calcrl^flf/fl^* mice (n = 8) and *Calcrl^fl/fl^/CAGGCre-ER™* mice (n = 9). **G,** Eye diagram indicating the location of components of the eye (l = lens, c = cornea, ac = anterior chamber, cb = ciliary body, i = iris). **H,** Lymphatic markers expressed in the eye shown by podoplanin(red) and Lyve-1(green) staining in a control mouse eye (20x objective, scale = 100 µm). **I,J,** Visualization of lymphatic vessels at the corneoscleral junction in the *Calcrl^flf/fl^* (I) and *Calcrl^fl/fl^/CAGGCre-ER™* mice (J) indicating enlarged lymphatic vessels in *Calcrl^fl/fl^/CAGGCre-ER™* mice (Lyve-1 = green; DAPI = blue; 20x objective, scale = 100 µm). **K,** Graph representing increased lymphatic vessel area at the corneoscleral junction in *Calcrl^fl/fl^/CAGGCre-ER™* mice compared to control mice calculated using Image J software(*p<0.015). Mice used were 3–4 months of age.

Tamoxifen injection resulted in a significant reduction of *Calcrl* gene expression in *Calcrl^fl/fl^/CAGGCre-ER™* animals compared to *Calcrl^fl/fl^* animals and to *Calcrl^fl/fl^/CAGGCre-ER™* non-injected animals (control animals) as indicated by qRT-PCR of lung and heart tissue (Figure S2). Within 7 to 10 days of tamoxifen injection, the majority of *Calcrl^fl/fl^/CAGGCre-ER™* mice, but none of the control *Calcrl^fl/fl^* tamoxifen-injected mice, developed distinct graying of their eyes and the surface of their corneas appeared rough and coarse (Figure 1A,B). The rapid onset of this phenotype prompted us to determine whether it was associated with glaucoma, since a previous study by Ittner et al. indicated that overexpression of *Calcrl* in smooth muscle of mice resulted in a phenotype similar to glaucoma [29]. To this end, TUNEL staining indicated no difference in retinal ganglion cell death between *Calcrl^fl/fl^/CAGGCre-ER™* and *Calcrl^fl/fl^* control mice (Figure S1A and S1B). We also found no significant histological differences in the optic nerve of *Calcrl^fl/fl^/CAGGCre-ER™* mice compared to *Calcrl^fl/fl^* control mice (data not shown). Finally, we found no significant difference in the intraocular pressure of *Calcrl^fl/fl^/CAGGCre-ER™* and *Calcrl^fl/fl^* control mice when compared either before injection or after injection of tamoxifen (Figure S1C) and all intraocular pressure measurements were within the normal range for C56BL/6 mice [22]. Taken together, these data rule out the possibility that the acute-onset eye phenotype in *Calcrl^fl/fl^/CAGGCre-ER™* mice is associated with classical features of glaucoma.

However, hematoxylin and eosin staining of eyes revealed marked changes in histology of the *Calcrl^fl/fl^/CAGGCre-ER™* corneas relative to those of *Calcrl^fl/fl^* control mice. The corneas of *Calcrl^fl/fl^/CAGGCre-ER™* mice were thickened and edematous and often showed a disrupted and damaged epithelial lining (Figure 1 C,D, arrow). We also observed pronounced inflammation in the anterior chamber and cornea of *Calcrl^fl/fl^/CAGGCre-ER™* mice (Figure 1E,F, arrowhead).

Based on the well-established role of *Calcrl* in lymphatic vascular development [12], the edema and inflammation in the corneas of *Calcrl^fl/fl^/CAGGCre-ER™* mice suggested to us that there may be problems with the lymphatic vessels of the eyes, particularly those within the corneoscleral junction, which is analogous to the conjunctival lymphatics in humans. Specifically, we found podoplanin-positive and LYVE-1-positive staining in the ciliary body and in vessels at the corneoscleral junction (Figure 1G,H, arrow). Interestingly, the lymphatic vessels at the corneoscleral junction of *Calcrl^fl/fl^/CAGGCre-ER™* mice were significantly dilated and twice the size of corneoscleral lymphatics in *Calcrl^fl/fl^* control mice (Figure 1I,J,K), similar to the phenotype observed in humans with conjunctival lymphangiectasia. More importantly, the eyes of *Calcrl^fl/fl^/CAGGCre-ER™* mice that did not present with the overt corneal pathology (approximately 1/3^rd^ of the mice), either because they failed to develop the phenotype or they were euthanized prior to the onset of the phenotype, still showed significantly dilated lymphatics at the corneoscleral junction. Taken together, these data demonstrate that an abnormal lymphatic vessel phenotype precedes the onset of acute corneal pathology in *Calcrl^fl/fl^/CAGGCre-ER™* mice.

### *Calcrl^fl/fl^/CAGGCre-ER™* Mice Exhibit Enlarged Submucosal Lymphatic Vessels and Lacteals in the Intestine with Dysfunctional Mesenteric Collecting Lymphatic Vessels

**Figure 2:**
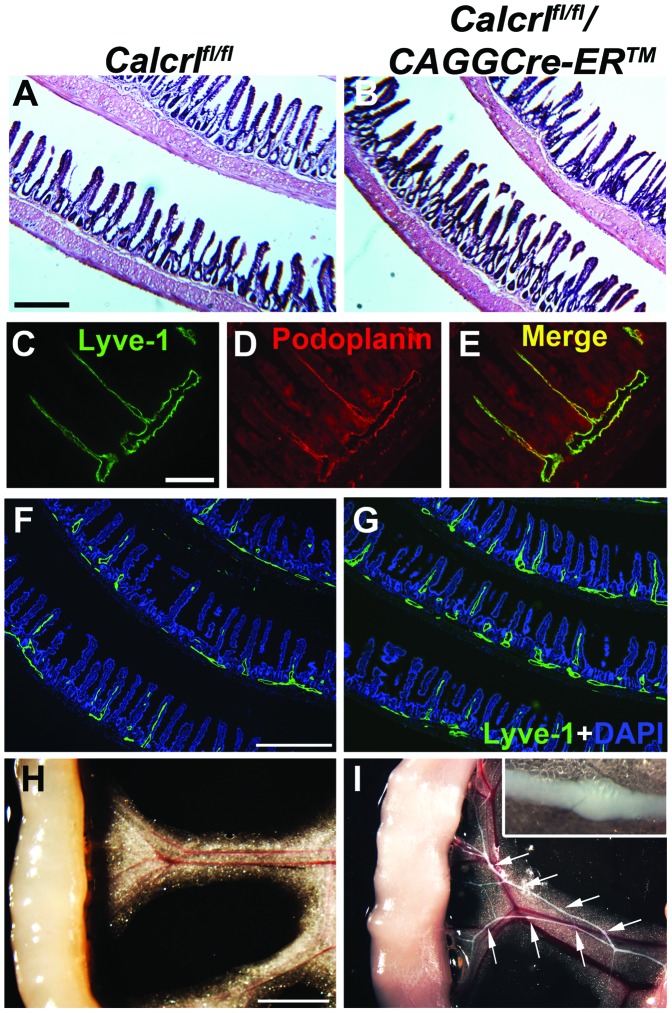
Dilated lacteals and submucosal lymphatics in *Calcrl^fl/fl^/CAGGCre-ER™* mice and chyle-filled lymphatics after short-term Western diet. A,B, Hematoxylin and eosin staining of mouse intestine showing normal histology in both *Calcrl^fl/fl^*(A) and *Calcrl^fl/fl^/CAGGCre-ER™* mice(B) (6.3x objective, scale = 500 µm). **C,D,E,** Lymphatic marker expression in the lacteals and submucosal lymphatic vessels in wildtype mouse. Image was obtained from the jejunum of the intestine. Lyve-1 (C,green) and podoplanin(D,red) colocalize in the lymphatic vessels as seen in the merged image(E) (20x objective; scale = 100 µm). **F,G** Lyve-1(green) and DAPI(blue) staining in *Calcrl^fl/fl^*(F) and *Calcrl^fl/fl^/CAGGCre-ER™* (G) mice indicating dilated lacteals and submucosal lymphatic vessels with temporal deletion of *Calcrl* in the jejunum of the intestine (4x objective, scale = 500 µm). Histology and immunofluorescent images are representative from *Calcrl^flf/fl^* mice (n = 7) and *Calcrl^fl/fl^/CAGGCre-ER™* mice (n = 6). **H,I,** Chyle-filled mesenteric collecting lymphatic vessels in *Calcrl^fl/fl^/CAGGCre-ER™* mice (I) relative to non-chyle filled vessels in control animals (H). Valves are distinctly visible in *Calcrl^fl/fl^/CAGGCre-ER™* mice (arrows; inset refers to enlarged image of valve; scale = 3 mm; n = 4 per genotype). Mice used were 6–8 months of age.

Since dilated lymphatic vessels were observed at the corneoscleral junction in the *Calcrl^fl/fl^/CAGGCre-ER™* mice, we wanted to assess the morphology and function of lymphatic vessels in other lymphatic vascular beds, for example, within the intestine. The overall histology of the intestines of *Calcrl^fl/fl^/CAGGCre-ER™* mice was normal when compared to that of *Calcrl^fl/fl^* control mice under normal conditions (Figure 2A,B). Lymphatic vessels within the intestine were identified with LYVE-1 and podoplanin staining, showing co-localization within the lacteals and the submucosal lymphatics (Figure 2C–E). Interestingly, the submucosal lymphatics and lacteals of the jejunum were also markedly dilated in the *Calcrl^fl/fl^/CAGGCre-ER™* mice (Figure 2F,G), as evidenced by the visibly larger diameter of the submucosal lymphatics and a greater proportion of villi sections revealing enlarged, LYVE-1-positive lacteals. Once again, these dilated lymphatics vessels are reminiscent of the dilated lymphatics observed in human patients with intestinal lymphangiectasia.

Intestinal lymphatics are required for normal lipid absorption, and patients with intestinal lymphangiectasia often present with weight loss as a result of lipid malabsorption [30]. Therefore, the function of these vessels was evaluated by placing *Calcrl^fl/fl^/CAGGCre-ER™* and *Calcrl^fl/fl^* control mice on a short term Western Diet following an overnight fast. After 1½ hours of Western Diet, the *Calcrl^fl/fl^/CAGGCre-ER™* mice exhibited chyle-filled mesenteric lymphatic vessels which were not visible in the *Calcrl^fl/fl^* control mice (Figure 2H,I). Chyle-filled submucosal lymphatic vessels were also visibly distinguishable in *Calcrl^fl/fl^/CAGGCre-ER™* mice and contributed to the whitened appearance of the intestine (Figure 2I), and some animals additionally exhibited chyle leakage into the mesenteric space. Importantly, the lymphatic valves of *Calcrl^fl/fl^/CAGGCre-ER™* mice appeared normal and were present at regular intervals along the mesenteric collecting lymphatics (Figure 2I, arrows and inset). Therefore, although the intestinal lymphatic vessels of *Calcrl^fl/fl^/CAGGCre-ER™* mice were present and appeared overtly normal, the collecting vessels were significantly dysfunctional in their transport of chyle as compared to control mice.

### Reduced Body Weight and Impaired Lipid Absorption with Protein-losing Enteropathy in *Calcrl^fl/fl^/CAGGCre-ER™* Mice

**Figure 3:**
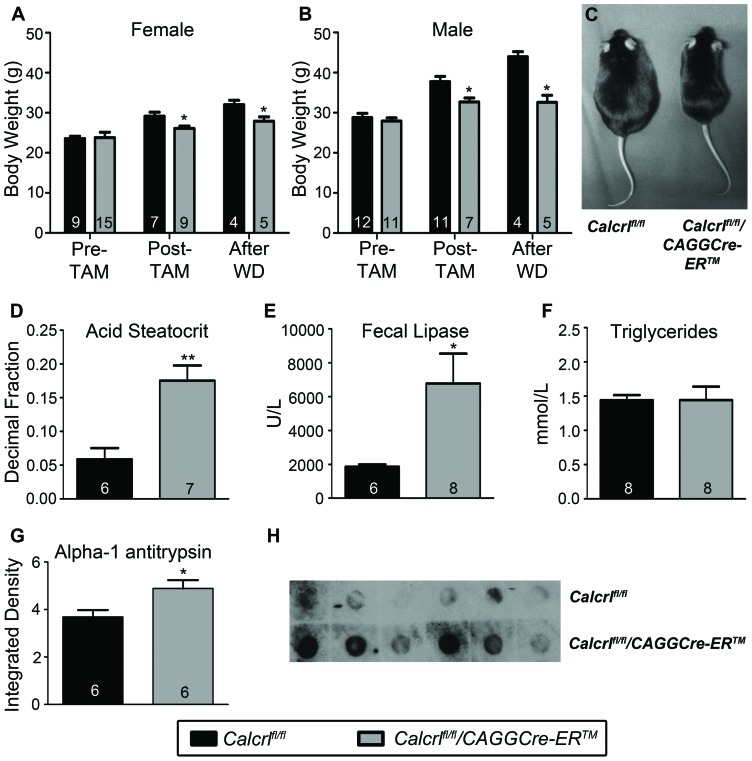
*Calcrl^fl/fl^/CAGGCre-ER™* mice exhibit reduced body weight due to impaired lipid absorption. A,B, Graphs of female(A) and male(B) body weights before injection of tamoxifen (Pre-TAM; 3–4 months of age), after injection of tamoxifen (Post-TAM; 3–4 months after), and after 1½ weeks on Western Diet (After WD). Both male and female *Calcrl^fl/fl^/CAGGCre-ER™* mice were significantly smaller than *Calcrl^fl/fl^* mice after tamoxifen injection and after Western Diet. **C,** Image of *Calcrl^fl/fl^* and *Calcrl^fl/fl^/CAGGCre-ER™* mice after 1½ weeks Western Diet. **D,** Acid steatocrit measurement in fecal samples from *Calcrl^fl/fl^* and *Calcrl^fl/fl^/CAGGCre-ER™* mice after Western Diet for 1½ weeks indicating increased lipid excretion in the experimental mice. **E,** Lipase measurements in fecal samples from *Calcrl^fl/fl^* and *Calcrl^fl/fl^/CAGGCre-ER™* mice on Western Diet for 1 ½ weeks indicating increased fecal lipase in experimental mice. **F,** Total triglyceride levels in *Calcrl^fl/fl^* and *Calcrl^fl/fl^/CAGGCre-ER™* mice. **G,** Alpha-1 antitrypsin levels in fecal samples from *Calcrl^fl/fl^* and *Calcrl^fl/fl^/CAGGCre-ER™* mice after 1½ weeks Western diet indicating lower levels in *Calcrl^fl/fl^/CAGGCre-ER™* mice. (Integrated density values are scaled and should be multiplied by 10^5^). **H,** Image of dot blot assay for alpha-1 antitrypsin in *Calcrl^fl/fl^* and *Calcrl^fl/fl^/CAGGCre-ER™* mice fecal samples after Western diet (1∶2000 dilution of samples). (*p<0.03;**p<0.002).

We next wanted to assess the impact of a longer term high fat diet on intestinal lipid absorption in the *Calcrl^fl/fl^/CAGGCre-ER™* mice. There were no significant differences in body weights between 3–4 month old, male or female *Calcrl^fl/fl^/CAGGCre-ER™* mice and *Calcrl^fl/fl^* control mice before the injection of tamoxifen (Figure 3A,B). However, 3–4 months after the injection of tamoxifen, we found that the *Calcrl^fl/fl^/CAGGCre-ER™* mice weighed significantly less than their control counterparts (Figure 3A,B), indicating that the tamoxifen-induced loss of *Calcrl* contributes to a failure of *Calcrl^fl/fl^/CAGGCre-ER™* to gain weight and thrive. The failure of *Calcrl^fl/fl^/CAGGCre-ER™* mice to gain weight and thrive was significantly exacerbated (Figure 3A,B) and visibly apparent (Figure 3C) when the mice were fed a Western Diet for 1½ weeks.

Moreover, fecal acid steotcrit levels, representative of lipid excretion levels, were significantly elevated in *Calcrl^fl/fl^/CAGGCre-ER™* animals maintained on a Western Diet for 1½ weeks compared to similarly fed *Calcrl^fl/fl^* control animals (Figure 3D), demonstrating reduced lipid absorption. Consistently, fecal pancreatic lipase levels were also increased in *Calcrl^fl/fl^/CAGGCre-ER™* animals, likely due to the compensatory effects of pancreatic lipase conversion of triglycerides into monoglycerides and free fatty acids during periods of reduced lipid absorption (Figure 3E). Importantly, levels of circulating triglycerides were unchanged in the *Calcrl^fl/fl^/CAGGCre-ER™* mice compared to *Calcrl^fl/fl^* control animals (Figure 3F), indicating that overall metabolism is not compromised in *Calcrl^fl/fl^/CAGGCre-ER™* mice and supporting the conclusion that their failure to gain weight is due to abnormal lipid absorption in the intestine. Finally, fecal samples of Western Diet-fed *Calcrl^fl/fl^/CAGGCre-ER™* mice contained a significantly elevated level of alpha-1 antiptrysin–a clinical diagnostic marker for protein-losing enteropathy–compared to similarly fed *Calcrl^fl/fl^* control mice (Figure 3G,H).

### Temporal Deletion of *Calcrl* Results in Increased Dermal Lymphatic Capillaries with Exacerbated and Prolonged Edema

**Figure 4:**
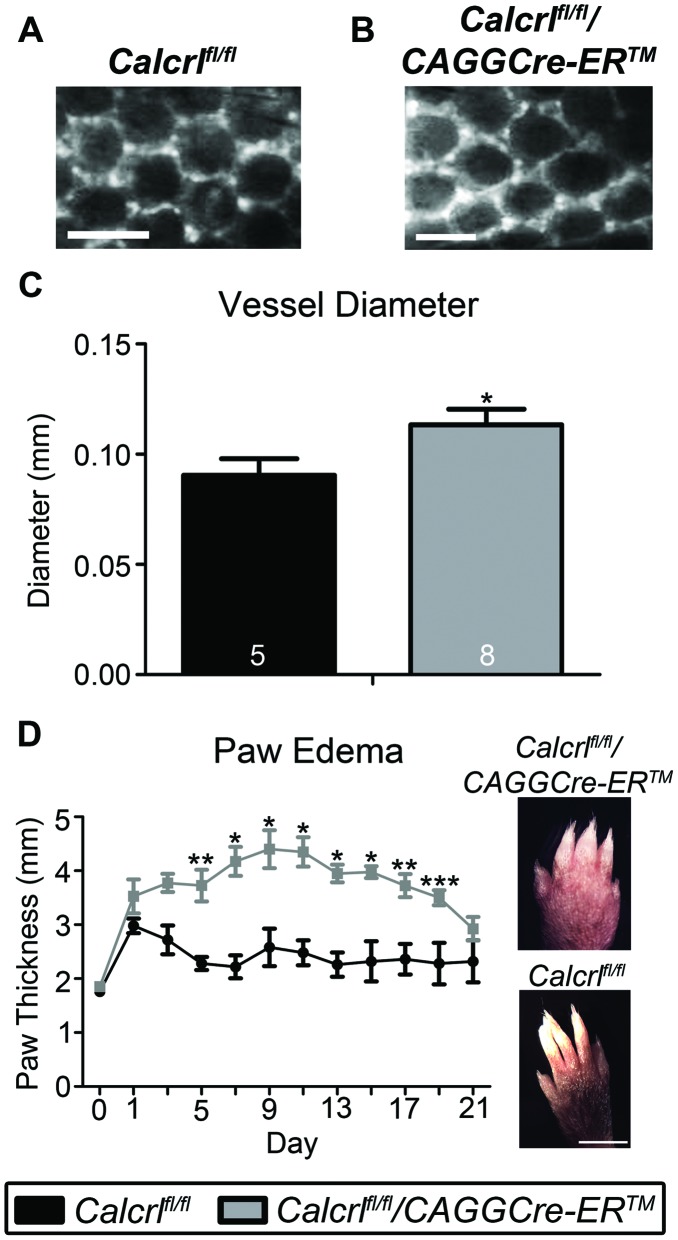
Dilated dermal lymphatic capillaries with exacerbated and prolonged edema. A,B, Images of dermal lymphatic capillaries in the tail of *Calcrl^fl/fl^*(A) and *Calcrl^fl/fl^/CAGGCre-ER™* (B) mice indicating increased diameter of these lymphatic vessels in *Calcrl^fl/fl^/CAGGCre-ER™* mice (scale = 0.5 mm). **C,** Graphic representation of the increase in vessel diameter in the *Calcrl^fl/fl^/CAGGCre-ER™* mice with respect to *Calcrl^fl/fl^* mice (*p≤0.05). **D,** Edema formation assay using hindpaw injections of CFA (4 µg/µl on Day 0). Assessment of paw thickness over 3 weeks (n = 5 for *Calcrl^fl/fl^* and n = 4 for *Calcrl^fl/fl^/CAGGCre-ER™* mice) indicated enhanced and prolonged edema in *Calcrl^fl/fl^/CAGGCre-ER™* mice relative to control mice (***p<0.05, **p<0.01, *p<0.001). Representative images of CFA-injected hindpaws at Day 11 for *Calcrl^fl/fl^* and *Calcrl^fl/fl^/CAGGCre-ER™* mice (scale = 3 mm). Mice used were 6–8 months of age.

**Figure 5:**
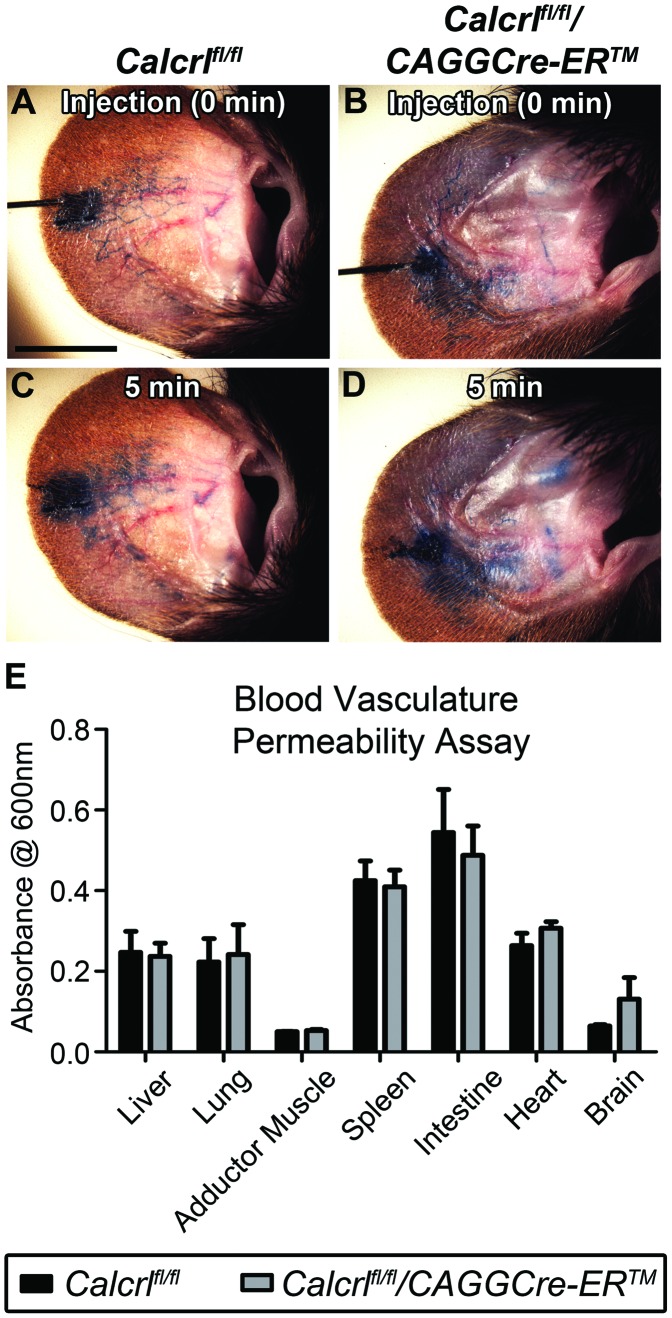
Increased lymphatic vascular permeability without change to blood vascular permeability in *Calcrl^fl/fl^/CAGGCre-ER™* mice. A,B,C,D, *In vivo* lymphatic permeability assay assessing the leakage of Evan’s blue dye from the dermal lymphatic vessels in the ear. Images represent Evan’s blue dye location directly after injection of the dye and 5 minutes post injection. There is an increase in leakage of the dye from the *Calcrl^fl/fl^/CAGGCre-ER™* mice (B,D) relative to *Calcrl^fl/fl^* mice (A,C). Depicted are representative images from four independent experiments (mice 6–8 months of age). **E,** Blood vascular permeability assay indicating there is no difference in permeability between genotypes in the various tissues (n = 4 per genotype for each tissue).

The dermal lymphatic capillaries of *Calcrl^fl/fl^/CAGGCre-ER™* mice also exhibited significant dilation and dysfunction. Specifically, intradermal injection of a large molecular weight (200 kDa) FITC-dextran into the subdermal area of the tail tip revealed significantly enlarged dermal capillaries in *Calcrl^fl/fl^/CAGGCre-ER™* mice compared to *Calcrl^fl/fl^* control mice (Figure 4A,B,C). Despite this dermal lymphangiectasia, we noticed that at the basal or quiescent state, *Calcrl^fl/fl^/CAGGCre-ER™* mice did not exhibit pronounced edema in their extremities (Figure 5F, day 0). Thus, we injected the hindpaws of *Calcrl^fl/fl^/CAGGCre-ER™* and *Calcrl^fl/fl^* control mice with (CFA) in order to challenge the lymphatic vascular system with localized edema. Both *Calcrl^fl/fl^/CAGGCre-ER™* mice and *Calcrl^fl/fl^* control mice exhibited a rapid and significant increase in hindpaw thickness within 1 day of CFA injection (Figure 4D). However, unlike the *Calcrl^fl/fl^* control mice which immediately began to resolve their edema by day 3, the *Calcrl^fl/fl^/CAGGCre-ER™* mice developed exacerbated and prolonged edema that peaked between days 9–11, and only began to show slight improvement after two weeks following CFA injection (Figure 4D). These data demonstrate that the expression of *Calcrl* is required for maintaining highly effective lymphatic function under conditions of edema and inflammation.

### *Calcrl^fl/fl^/CAGGCre-ER™* Mice Exhibit Increased Lymphatic Capillary Permeability with No Apparent Disruption of Blood Vascular Permeability

To better evaluate the permeability of lymphatic and blood vasculatures in *Calcrl^fl/fl^/CAGGCre-ER™* and *Calcrl^fl/fl^* mice, we used the small molecular weight Evan’s blue dye which can freely penetrate in and out of dermal capillaries. Injection of 0.5% Evan’s blue dye intradermally in the ear showed rapid uptake of the dye by dermal lymphatics in both *Calcrl^fl/fl^/CAGGCre-ER™* and *Calcrl^fl/fl^* mice (Figure 5A,B). However after 5 minutes, *Calcrl^fl/fl^/CAGGCre-ER™* mice exhibited increased leakage of the dye from the lymphatic vessels, as evidenced by the diffuse spreading of the dye and poorly demarcated lymphatics throughout the ear region compared to the *Calcrl^fl/fl^* control mice (Figure 5C,D). To determine whether this lymphatic permeability defect was impacted or perhaps confounded by a permeability defect in the blood vasculature, we also measured relative blood vascular permeability in mice receiving a venous injection of Evan’s blue dye. Absorbance readings of Evan’s blue dye showed no statistically significant differences between *Calcrl^fl/fl^/CAGGCre-ER™* mice and *Calcrl^fl/fl^* control mice for multiple tissues including liver, lung, adductor muscle, spleen, intestine, heart and brain (Figure 5E). Based on these data, we conclude that temporal deletion of *Calcrl* in adult animals results in increased lymphatic capillary permeability with no overt or functional changes in blood vascular permeability.

### Inhibition of AM Signaling Results in Disorganization of Lymphatic Endothelial Cell Junctions

**Figure 6:**
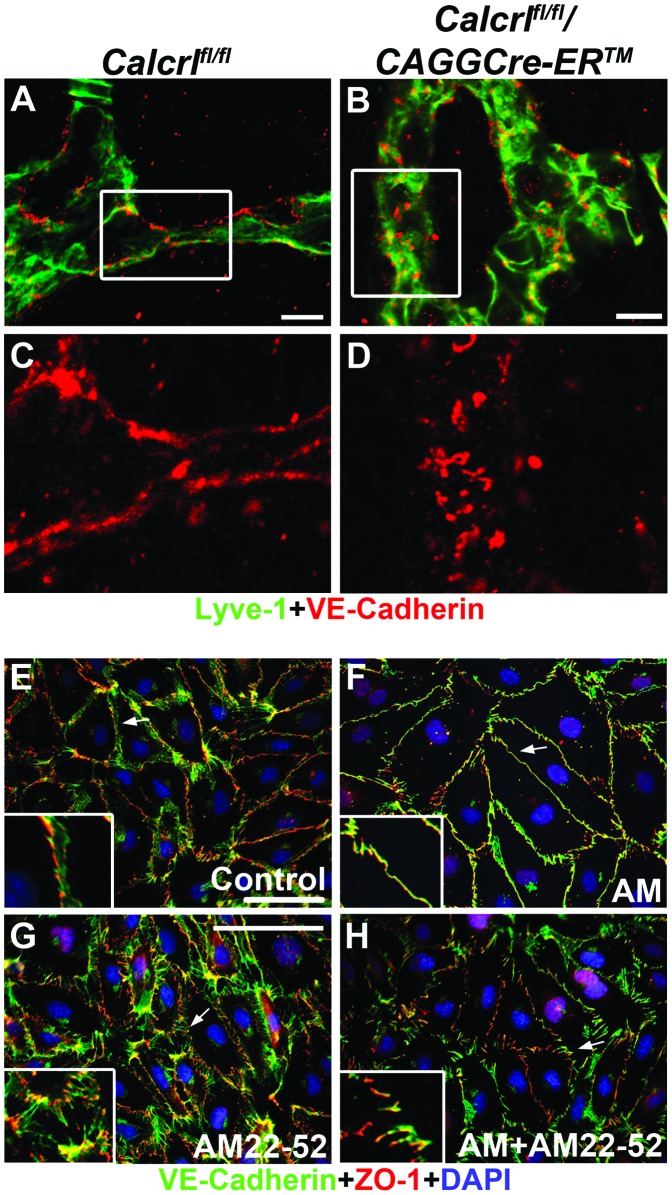
Inhibition of AM signaling disrupts lymphatic endothelial cell-cell junctions. A,B,C,D, Confocal images of VE-Cadherin (red) and Lyve-1(green) expression in mesenteric lymphatic vessels of *Calcrl^fl/fl^* (A,C) and *Calcrl^fl/fl^/CAGGCre-ER™* mice (B,D) (scale = 10 µm). Boxed region depicted in C and D. Junctional protein, VE-cadherin, is disorganized in *Calcrl^fl/fl^/CAGGCre-ER™* mice relative to *Calcrl^fl/fl^* mice (representative images from n = 4 per genotype, age 6–8 months). **E,F,G,H,** Lymphatic endothelial cells stained with VE-Cadherin (green), ZO-1 (red) and DAPI (blue) after various treatments including a no treatment control (A), 10 nm AM (B), 1 µm AM22-52 (C), AM+AM22-52 (D) (arrow refers to inset region). Disorganization of cell-cell junctions occurs with inhibitor treatment (AM22-52) as compared to AM treatment. (VE-cadherin = red, Lyve-1 = green, DAPI = blue, 40x objective, scale = 100 µm; representative images from 3 independent experiments).

To elucidate the molecular mechanisms contributing to the lymphatic dysfunction in *Calcrl^fl/fl^/CAGGCre-ER™* mice, we evaluated VE-Cadherin expression and localization in mesenteric lymphatic vessels of *Calcrl^fl/fl^/CAGGCre-ER™* and *Calcrl^fl/fl^* control mice that had been fed a high fat diet for 1½ hours. VE-Cadherin expression was visibly disrupted in lymphatic vessels of *Calcrl^fl/fl^/CAGGCre-ER™* mice (Figure 6B,D) compared to control mice (Figure 6A,C). More specifically, while the relative expression levels of VE-cadherin appeared similar between genotypes, the VE-cadherin in mesenteric lymphatic vessels of *Calcrl^fl/fl^/CAGGCre-ER™* appeared as punctate lobules throughout the cells and was not localized to well-defined cell boundaries, as seen in the *Calcrl^fl/fl^* control mice.

To better characterize the effects of inhibiting *Calcrl*-mediated signaling in cultured lymphatic endothelial cells (LECs) we used an adrenomedullin-specific peptide inhibitor, AM22-52. As we have previously described, treatment of LECs with AM peptide resulted in a marked reorganization of junctional proteins, VE-Cadherin and ZO-1, from a jagged, zipper-like configuration to a cohesive and stabilized cell-cell barrier that is functionally associated with reduced permeability (Figure 6E,F and [17]). In contrast, treatment with the *Calcrl* antagonist, AM22-52, either alone or in combination with AM, abolished the effects of AM peptide and resulted in highly disorganized and jagged junctional protein configurations (Figure 6G,H). Taken together, these results demonstrate that *in vitro* and *in vivo* inhibition of *Calcrl* signaling, either by antagonist treatment or by genetic deletion, results in a profound loss of junctional protein organization, likely resulting in increased permeability of lymphatic endothelial cell barriers.

## Discussion

These studies demonstrate that temporal loss of murine *Calcrl* in adulthood causes lymphatic insufficiency in a wide range of organs, representing functional similarities to the sequelae observed in patients with a variety of lymphangiectasia conditions. Consistently, the lymphatic vessels in the eye, intestine and skin of *Calcrl^fl/fl^/CAGGCre-ER™* mice were dilated, had irregular junctional protein organization and were dysfunctional when challenged with either fat absorption or edema and inflammation. Taken together, these data identify an important new role for AM signaling as a potent regulator of lymphatic vascular drainage and permeability in adult animals.

The rapid-onset eye phenotype in *Calcrl^fl/fl^/CAGGCre-ER™* mice provides novel and clinically relevant insights to the potential role of lymphatic vessels in the eye. Several recent studies have shown that lymphatic markers are expressed in the human eye [31], [32], but it is still unclear whether and how these lymphatic vessels contribute to fluid homeostasis of the eye. Our staining of lymphatic markers in the eye correlates well with these previous studies, since we showed robust LYVE-1 and podoplanin staining in the ciliary body and distinct LYVE-1-positive lymphatic vessels in the corneoscleral junction. Most importantly, we found that temporal deletion of *Calcrl* resulted in dilated corneoscleral lymphatic vessels that preceded and were associated with the formation of corneal edema and inflammation. Therefore, it is likely that appropriate fluid homeostasis and hydration of the cornea, which is an important physiological feature to consider in terms of dry eye disease, corneal surgeries or conjunctival lymphangiectasia, is modulated by lymphatic vessels. Since AM peptide can be clinically administered [33] and the *Calcrl/Ramp* interface is pharmacologically tractable [34], [35], the potential of harnessing these targets for the therapeutic modulation of fluid homeostasis in the eye may prove to be an exciting avenue.

The intestinal lymphatic phenotype of *Calcrl^fl/fl^/CAGGCre-ER™* mice also correlates well with the clinical presentation of intestinal lymphangiectasia in humans. Under a short-term Western Diet, *Calcrl^fl/fl^/CAGGCre-ER™* mice showed signs of lymphatic insufficiency because their intestinal mesenteric lymphatic vessels failed to transport chyle as effectively as similarly fed control mice. Because *Calcrl^fl/fl^/CAGGCre-ER™* mice are significantly leaner than age-matched control mice 3–4 months post tamoxifen injection, it is likely that the collecting mesenteric lymphatics of these animals function at a consistently reduced capacity. In this regard, it is important to note that weight loss is often associated with lymphangiectasia in the form of protein-losing enteropathy [5] and lipid malabsorption. Consistently, the *Calcrl^fl/fl^/CAGGCre-ER™* mice also exhibit elevated alpha-1 antitrypsin in fecal samples after Western diet, which is indicative of protein-losing enteropathy, similar to the clinical phenotype that is frequently observed in humans with intestinal lymphangiectasia. While the mechanism of lipid absorption through lymphatic lacteals is not completely understood, it is thought to involve both active and passive transport mechanisms through lymphatic endothelial cells [36]. Our data demonstrate that AM signaling through *Calcrl/Ramp2* is required for normal intestinal lipid uptake and junctional protein organization in intestinal lymphatic capillaries. Whether the maintenance of the lymphatic permeability barrier and loss of *Calcrl* is connected with the active and/or passive transport mechanism of lipid absorption within the lacteal will be an important future area of study that may have bearing on better understanding the functional underpinnings of intestinal lymphangiectasia.

It is notable that lymphangiectasia is commonly associated with limb edema. When placed under challenge, we found that the hindpaw of *Calcrl^fl/fl^/CAGGCre-ER™* mice had significantly exacerbated edema that resolved over a longer time period than similarly challenged wildtype animals. These results correlate with the results from the high fat diet in that the lymphatic system of the *Calcrl^fl/fl^/CAGGCre-ER™* mice does not respond effectively as that of control mice to different stresses indicating that there are dysfunctional lymphatic vessels in the *Calcrl^fl/fl^/CAGGCre-ER™* mice.

Importantly, the *Calcrl^fl/fl^/CAGGCre-ER™* mice do not exhibit overt edema in the basal state and we found no significant effects of *Calcrl* loss on blood vascular permeability. Studies by T. Shindo and colleagues using *Ramp2* gene targeted mice suggested that loss of *Ramp2* led to a reduction in the expression of junctional proteins and a loss of blood vascular integrity [37]. Using an independent line of *Ramp2* gene targeted mice, we have demonstrated that *Ramp2^−/−^* mice also have arrested lymphangiogenesis [12]. Because Ramp2 associates with multiple G protein-coupled receptors beyond *Calcrl*, it is likely that the expanded vascular phenotypes of *Ramp2^−/−^* mice can be attributed to additional signaling pathways, and this notion is further supported by the extensive endocrine phenotypes of *Ramp2^+/−^* mice compared to *Calcrl^+/−^* mice [38]. Taken together, these data continue to support a predominant and preferential role for *Calcrl* in the lymphatic vasculature compared to the blood vasculature [39], which may be partially explained by the fact that *Calcrl* and *Ramp2* are expressed at higher levels in lymphatic endothelial cells compared to blood endothelial cells [18]–[20].

*Calcrl* also serves as a receptor component for the neuropeptide, calcitonin gene related peptide (CGRP), when the receptor is associated with RAMP1. Therefore, we cannot formally exclude the possibility that the phenotypes from temporal loss of *Calcrl* are not partially attributable to loss of CGRP signaling. For example, other studies have indicated that adult αCGRP knockout mice fed a high fat diet do not gain as much weight as control mice. However, in contrast to the *Calcrl^fl/fl^/CAGGCre-ER™* mice, the αCGRP knockout mice eat more and have higher levels of energy expenditure compared to controls [43]. Also, in our colony, αCGRP knockout mice and RAMP1 knockout mice have never exhibited the visible eye phenotype that is hallmark of the *Calcrl^fl/fl^/CAGGCre-ER™* mice. Finally, the αCGRP knockout mice [40]–[42] are not embryonic lethal and no vascular defects have been reported in these mice. In contrast, many similarities exist between the phenotypes of *Calcrl* knockout mice and those of AM and RAMP2 knockout mice, with a primary defects in the vasculature. Therefore, while some implication of CGRP signaling cannot be excluded in the *Calcrl^fl/fl^/CAGGCre-ER™* mice, the phenotypes revealed are more consistent with a predominant attribution to AM signaling. Nevertheless, additional characterization of *Calcrl^fl/fl^/CAGGCre-ER™* mice for phenotypes more closely associated with the physiological functions of CGRP, like pain perception, will be an interesting future direction.

Ultimately these studies indicate functional similarities between temporal loss of *Calcrl* in adult mice and human lymphangiectasia, but the mechanistic relationship remains elusive and will be an interesting focus for future studies. The underlying cause of lymphatic insufficiency in *Calcrl^fl/fl^/CAGGCre-ER™* mice is likely attributable to various mechanisms including insufficient lymph transport and disrupted lymphatic vessel permeability. The *Calcrl^fl/fl^/CAGGCre-ER™* mice do not respond sufficiently to stress on the lymphatic system indicating the lymphatic network is dysfunctional. Moreover, our permeability findings are consistent with previous studies showing that addition of AM both *in vitro* and *in vivo* results in decreased permeability of LECs and lymphatic vessels through reorganization of the junctional proteins VE-cadherin and ZO-1 [17]. In the blood vasculature, Rap1 (Ras-related protein 1), a small GTPase, plays a predominant role in regulating cell adhesion and cell junction organization in response to cAMP/Epac/ERK signaling pathways [44]. Since the major downstream effectors of AM signaling in LECs are cAMP/Epac/ERK, it will be interesting in future studies to determine whether similar or identical Rap1 mechanisms contribute to the lymphatic permeability phenotypes of *Calcrl^fl/fl^/CAGGCre-ER™* mice.

## Supporting Information

Figure S1 **The acute onset eye phenotype with temporal deletion of** ***Calcrl*** **is not associated with glaucoma-like characteristics. A,B,** TUNEL staining of retinal ganglion cells (arrows) in *Calcrl^fl/fl^* (A) and *Calcrl^fl/fl^/CAGGCre-ER™* mice (B) (DAPI = red; TUNEL = green; scale = 100 µm) **C,** Tonometry measurements of intraocular pressure in *Calcrl^fl/fl^* and *Calcrl^fl/fl^/CAGGCre-ER™* mice before tamoxifen injection and one month post-injection.
(TIF)

Figure S2 ***Calcrl*** **gene expression in lung and heart tissue of** ***Calcrl^fl/fl^*** **and** ***Calcrl^fl/fl^/CAGGCre-ER™*** **. A,** qRT-PCR quantitation of relative expression of *Calcrl* normalized to mouse elongation factor in *Calcrl^fl/fl^* and *Calcrl^fl/fl^/CAGGCre-ER™* non-injected control mice relative to *Calcrl^fl/fl^/CAGGCre-ER™* mice. There is a significant reduction in *Calcrl* expression in both lung and heart tissue of *Calcrl^fl/fl^/CAGGCre-ER™* mice relative to control mice (*p<0.04, **p<0.02).
(TIF)
