# Supplementary figures and images for: Characteristics of Multi-Organ Lymphangiectasia Resulting from Temporal Deletion of Calcitonin Receptor-Like Receptor in Adult Mice
Source: PLoS One. 2012 Sep 17;7(9):e45261. doi: 10.1371/journal.pone.0045261 (PMC3444480; doi:10.1371/journal.pone.0045261)

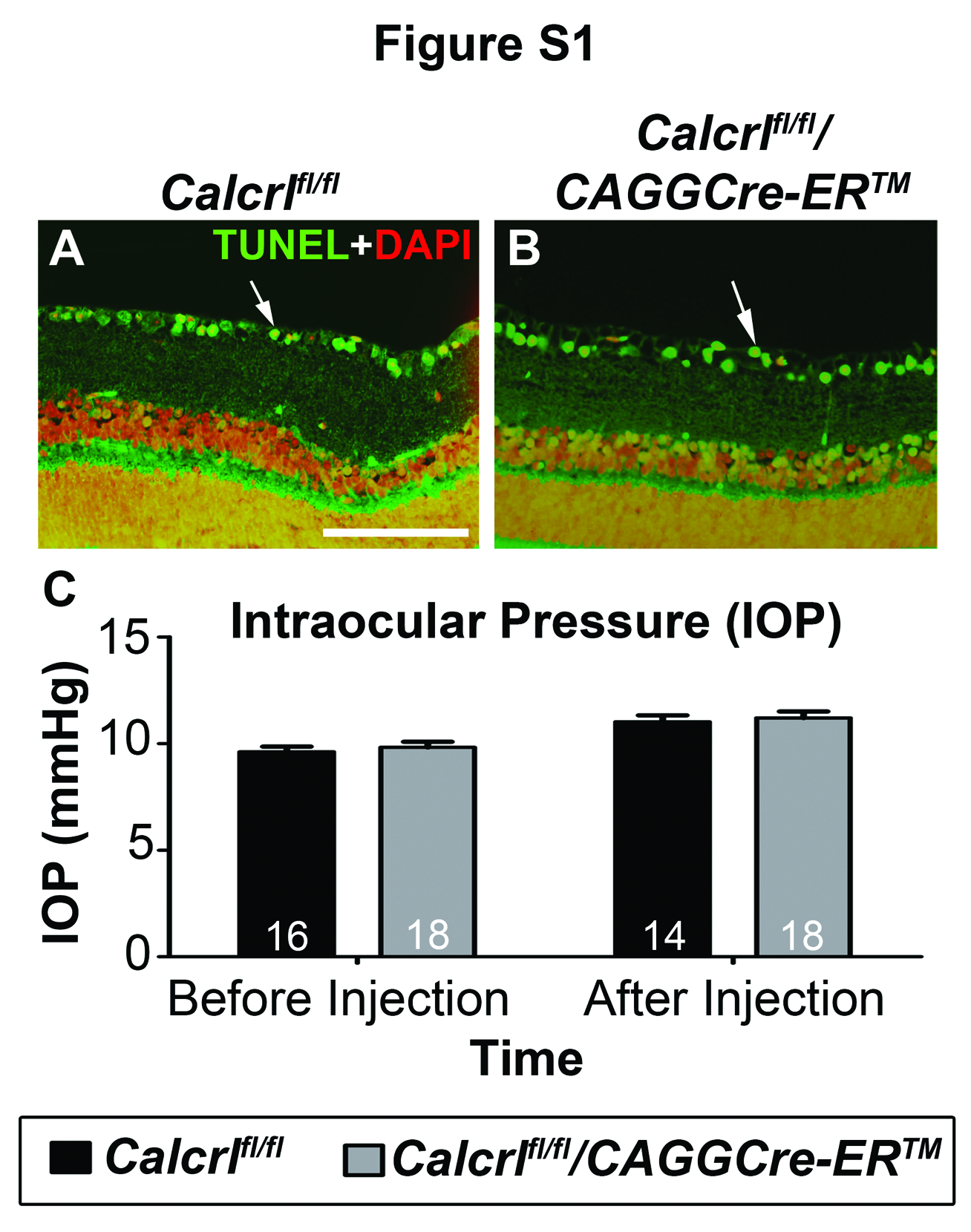

Supplement: Figure S1 — The acute onset eye phenotype with temporal deletion of Calcrl is not associated with glaucoma-like characteristics. A,B, TUNEL staining of retinal ganglion cells (arrows) in Calcrlfl/fl (A) and Calcrlfl/fl/CAGGCre-ER™ mice (B) (DAPI = red; TUNEL = green; scale = 100 µm) C, Tonometry measurements of intraocular pressure in Calcrlfl/fl and Calcrlfl/fl/CAGGCre-ER™ mice before tamoxifen injection and one month post-injection. (TIF) [file pone.0045261.s001.tif]

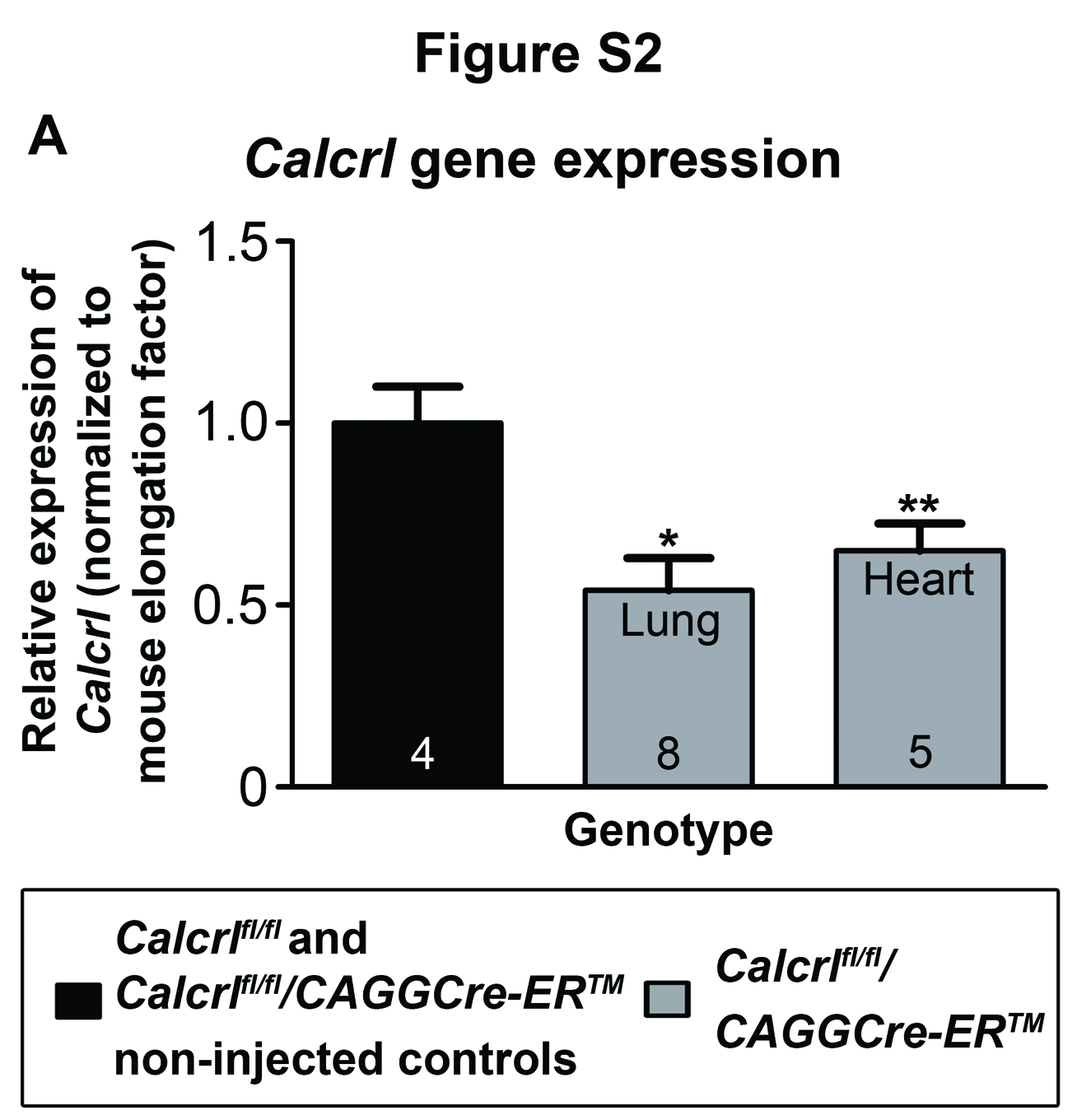

Supplement: Figure S2 — Calcrl gene expression in lung and heart tissue of Calcrlfl/fl and Calcrlfl/fl/CAGGCre-ER™ . A, qRT-PCR quantitation of relative expression of Calcrl normalized to mouse elongation factor in Calcrlfl/fl and Calcrlfl/fl/CAGGCre-ER™ non-injected control mice relative to Calcrlfl/fl/CAGGCre-ER™ mice. There is a significant reduction in Calcrl expression in both lung and heart tissue of Calcrlfl/fl/CAGGCre-ER™ mice relative to control mice (*p<0.04, **p<0.02). (TIF) [file pone.0045261.s002.tif]
